# Differential levels of L-homocysteic acid and lysophosphatidylcholine (16:0) in sera of patients with ovarian cancer

**DOI:** 10.3892/ol.2014.2214

**Published:** 2014-06-03

**Authors:** SEUNG CHEOL KIM, MIN KYUNG KIM, YUN HWAN KIM, SUN-A AHN, KYUNG-HEE KIM, KUN KIM, WON KI KIM, JUN HWA LEE, JAE YOUL CHO, BYONG CHUL YOO

**Affiliations:** 1 Division of Gynecologic Oncology, Department of Obstetrics and Gynecology, Ewha Woman’s University Mokdong Hospital, Ewha Woman’s University School of Medicine, Seoul 158-710, Republic of Korea; 2 Colorectal Cancer Branch, Research Institute, National Cancer Center, Gyeonggi 410-769, Republic of Korea; 3 Department of Genetic Engineering, Sungkyunkwan University, Gyeonggi 440-746, Republic of Korea

**Keywords:** ovarian cancer, L-homocysteic acid, lysophosphatidylcholine (16:0), biomarker, cancer screening

## Abstract

Ovarian cancer (OVC) is one of the most difficult types of cancer to detect in the early stages of its development. There have been numerous attempts to identify a biomarker for OVC; however, an accurate diagnostic marker has yet to be identified. The present study profiled OVC candidate metabolites from the serum to identify potential diagnostic markers for OVC. Data regarding low-mass ions (LMIs) in the serum were obtained using matrix-assisted laser desorption/ionization (MALDI)-time-of-flight analysis. MALDI-mass spectrometry (MS) analysis of each serum sample was repeated six times in order to reduce the likelihood of experimental errors. The intensity of the LMI mass peaks were normalized using total peak area sums. The normalized intensity of LMI was used in principal component analysis-discriminant analysis to differentiate between 142 patients with OVC and 100 healthy control participants. Liquid chromatography-MS/MS was used to identify the selected LMIs. Extracted ion chromatogram analysis was used to measure the relative quantity of candidate metabolites from the LMI mass peak areas. The concentration of common metabolites in the serum was determined using ELISA. The top 20 LMI mass peaks with a weigh factor over 0.05 were selected to distinguish between the patients with OVC and the controls. Among the LMIs, two with 184.05 and 496.30 m/z were identified as L-homocysteic acid (HCA) and lysophosphatidylcholine (LPC) (16:0), respectively. The relative quantity of LPC (16:0) was found to be decreased in the OVC serum (P=0.05), while the quantity of HCA was observed to be significantly higher in the OVC serum (P<0.001). HCA was not detected in 59 cases out of the 63 control participants; however, the majority of the cases of OVC (16/25) exhibited significantly higher quantities of HCA. When the cutoff was 10 nmol/ml, the sensitivity and specificity of HCA were 64.0 and 96.9%, respectively. The level of LPC (16:0) was significantly correlated with tumor grade (P=0.045). HCA and LPC (16:0) showed correlation with stage and tumor histology, but the limited sample size resulted in a lack of statistical significance. The findings of the present study suggest that HCA may have potential to be a biomarker for OVC. The stratified screening including LPC (16:0) did not significantly increase the power for OVC screening; however, the present study showed that profiling LMIs in serum may be useful for identifying candidate metabolites for OVC screening.

## Introduction

Ovarian cancer (OVC) is one of the most frequently occurring types of gynecological cancer, with 204,000 new cases identified each year and a five-year survival rate of 44% for all stages of cancer development (1–4). More than 70% of OVC cases are identified in the late stages of cancer (stage III or IV according to the International Federation of Gynecology and Obstetrics standard) (2). Despite improvements in anticancer therapeutic methods, the mortality rate of OVC has not decreased over the past 20 years due to difficulties in screening early stages of the disease (5). Current diagnostic methods include pelvic examination, ultrasonograms, blood tests and tissue examination (6,7); however, these methods have several limitations, including their inability to diagnose OVC at an early stage or to detect invasiveness. Thus, early and easy-to-use diagnostic methods for OVC are required in order to increase the survival rate of patients with OVC.

Several previous studies have investigated the use of serological markers to accurately detect OVC. Such markers include cancer antigen (CA) 125, human epididymis protein 4 (HE4), and macrophage colony-stimulating factor (M-CSF) (5,8,10). Serum CA125 and HE4 concentrations have been used as markers for OVC using radioimmunoassay (6,9,10). Furthermore, 70% of patients with OVC with various OVC cell lines have high serum levels of M-CSF (11). While M-CSF is a monocyte-specific cytokine for proliferation and differentiation, it also acts as a growth factor for certain epithelial cancers in an autocrine and paracrine manner (12). However, these markers lack accuracy and have difficulty in early diagnosis. For example CA125 was discovered 20 years ago and has been used widely as an OVC marker since (13). However, CA125 has low specificity and sensitivity during the early stages of OVC (6,9,14), thus an ideal marker has yet to be elucidated.

The present study profiled low-mass metabolic compounds in methanol/chloroform extracts obtained from the sera of patients with OVC and healthy controls using matrix-assisted laser desorption/ionization-time-of-flight (MALDI-TOF) mass spectrometry and identified two molecules using tandem mass spectrometry (MS/MS) analysis. The present study identified a differential pattern of lysophosphatidylcholine (LPC) (16:0) and L-homocysteic acid (HCA) in patients with OVC, and discusses the advantages of profiling low-mass metabolic compounds for screening OVC.

## Materials and methods

### Serum from patients with OVC

All participants provided written informed consent and the study protocol was approved by the Institutional Review Board of the Ewha Womans University (Seoul, Korea). A total of 142 patients and 100 control participants were enrolled in the present study (Table I).

### MALDI-TOF analysis for collecting low-mass ions (LMIs) in serum

Four times volume of methanol/chloroform (2:1; v/v) was incubated with 25 μl serum for 10 min at room temperature subsequent to vortexing. The solution was centrifuged at 6,000 × g for 10 min at 4°C. The supernatant was then dried in a concentrator for 1 h and resolved in 30 μl 50% acetonitrile/0.1% trifluoroacetic acid (TFA) using a vortex for 30 min.

Methanol/chloroform extract was mixed (1:12; v/v) with an α-cyano-4-hydroxycinnamic acid solution in 50% acetonitrile/0.1% TFA. A total of 1 μl of the solution was then spotted on the MALDI target for analysis. Individual mass spectra from the serum extracts of the patients with OVC were obtained using a 4700 Proteomics Analyzer (Ab Sciex, Framingham, MA, USA). The mass-spectral data represent the average of 20 accumulated spectra. All individual peak areas were normalized to the total area up to 2,500 m/z. To minimize experimental error, variable factors including focus mass, laser intensity, target plate and data acquisition time were tested. The ideal focus mass and laser intensity were fixed at 500 m/z and 5,000, respectively (15). With the fixed focus mass and laser intensity, one sample was analyzed six times under the different extraction and data acquisition times.

### LMI selection and statistical analysis

All MALDI mass spectra, formatted as t2d files, were analyzed using MarkerView™ software, version 1.2 (Applied Biosystems/MDS Sciex, Toronto, ON, Canada). The optimized parameters used to compare LMI mass peaks in the serum extracts obtained from the patients with OVC were as follows: Mass tolerance, 100 ppm; minimum required response, 100; maximum number of peaks, 5000; and normalization, by total area sums. Subsequent to collecting the data using MALDI mass spectra, principal component analysis-discriminant analysis (PCA-DA) and t-tests were used to select LMIs with differential peak intensities in serum extracts from patients with OVC.

### Measurement of HCA in serum

The level of HCA in the sera was measured using an ELISA kit (Cusabio Biotech, Co., Ltd., Wuhan, China) according to the manufacturer’s instructions.

### Measurement of LPC (16:0) in serum

A nanoflow high-performance liquid chromatography instrument (Easy nLC; Thermo Scientific, Inc., Waltham, MA, USA) was coupled to an LTQ mass spectrometer (Thermo Scientific, Inc.). A PepMap^®^ RSLC, C18, 2 μm, 100 Å analytical column (50 cm; inner diameter, 75 μm; Dianex Corporation, Sunnyvale, CA, USA) was used. Reversed phase chromatography was performed using a binary buffer system consisting of 0.1% formic acid (buffer A) and acetonitrile in 0.1% formic acid (buffer B). The sample was separated using a linear gradient of 3–50% buffer B at a flow rate of 300 nl/min. The gradient time was 90 min and the total run time for the liquid chromatography MS/MS was 120 min. The extracted LPC was analyzed using the selected reaction monitoring (SRM) mode. The SRM transitions for the LPC lipid were set to m/z 496.4 to 183.96 and m/z 496.4 to 478.33. The SRM data were acquired within fragment ion mass ± 2 m/z and each SRM transition and respective retention time was validated for specific LPC. Data were processed through integrating the appropriate peaks for LPC, followed by comparing the calculated peak areas using two-paired t-tests.

### Statistical analysis

Between-group differences were calculated using the student’s t-test and within-group correlations were calculated using Spearman’s rank correlation coefficient. P<0.05 was considered to indicate a statistically significant difference.

## Results

### Differential LMIs in methanol/chloroform extracts from the sera of patients with OVC

**Figure 1:**
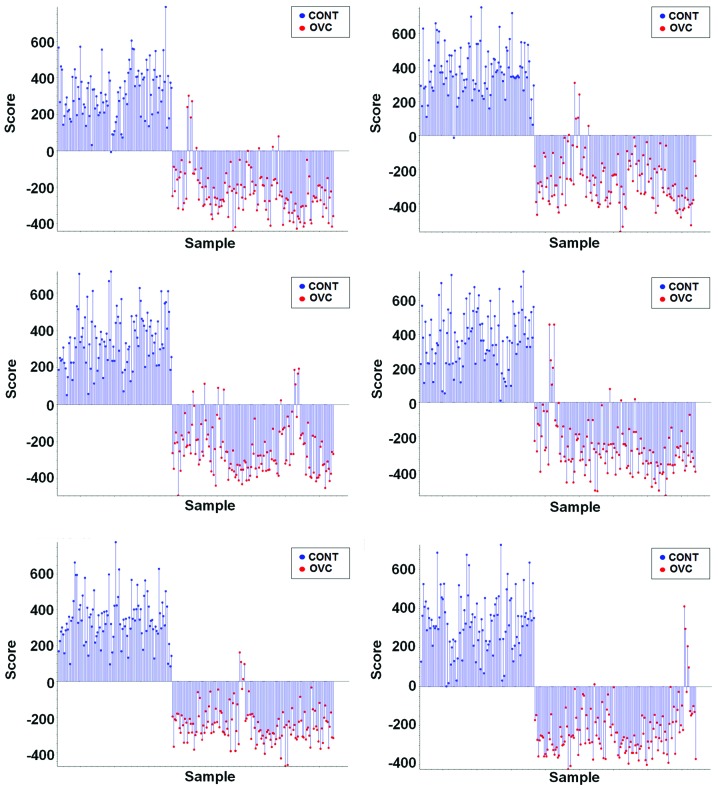
PCA-DA of the sera of 142 patients with OVC and 100 healthy controls. Methanol/chloroform extracts from the sera of 142 patients with OVC and 100 healthy controls were used for MALDI-TOF analysis. LMI data (m/z and mass peak intensity) from the extracts was obtained using MALDI-TOF analysis six times. The intensities of all of the individual LMIs were normalized using the ‘total peak area sums’. The m/z and normalized intensity of LMI was used in PCA-DA. Classification results of PCA-DA repeated six times reveal that the pattern of LMI in the sera of the patients with OVC was different to that of the controls. PCA-DA, principal component analysis-discriminant analysis; OVC, ovarian cancer; MALDI-TOF, matrix-assisted laser desorption/ionization-time-of-flight; LMI, low-mass ion; Cont, control.

Data (m/z and mass peak intensity) regarding the LMIs with mostly <1,000 m/z collected from the sera extracts of 100 healthy control individuals and 142 patients with OVC were used in the PCA-DA in order to determine whether differential LMI patterns exist in the sera of patients with OVC. Supervised PCA-DA using LMI data obtained from six repeats of MALDI-TOF analysis discriminated the patients with OVC from the control individuals (Fig. 1).

### Selection and identification of LMIs showing a differential pattern in patients with OVC

**Figure 2:**
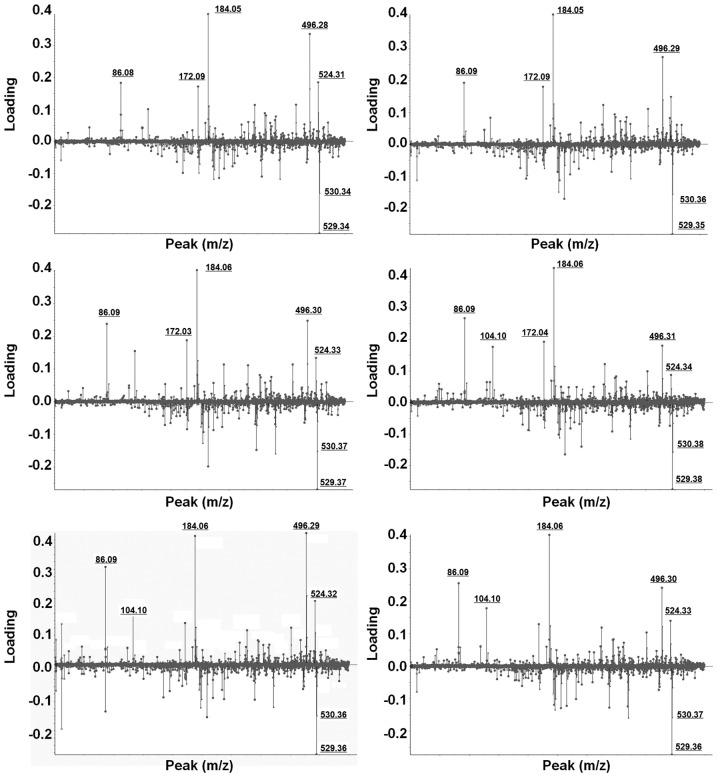
Selection of LMIs with higher weighting factors. Weighting factors (or loading) for individual LMIs were calculated using principal component analysis-discriminant analysis. LMIs showing a higher weighting factor in each independent analysis were selected for further identification and validation. There was a slight mass shifting in repeated matrix-assisted laser desorption/ionization-time-of-flight analyses. LMIs with 184.05 and 496.30 m/z showed strong and constant discriminating power for ovarian cancer screening. LMI, low-mass ion.

Weighting factors (loading value) for all individual LMIs were calculated using PCA-DA (Fig. 2). LMIs which consistently exhibited higher weighting factors in six different PCA-DA analyses were selected. Despite slight mass shifting, LMIs with 184.05 and 496.30 m/z showed strong discriminating power for OVC screening (Fig. 2).

**Figure 3:**
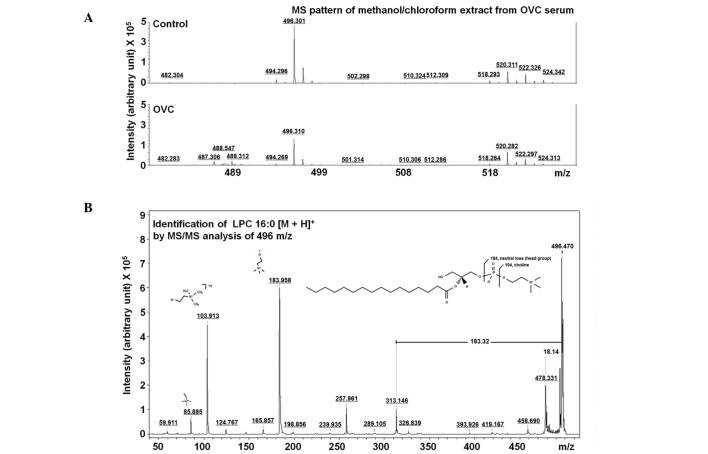
Identification of LMIs with 496.30 m/z. (A) Mass peak pattern of an LMI with 496.30 m/z on mass spectra. (B) MS/MS analysis for the identification of an LMI with 496.30 m/z. The MS/MS spectrum of the LMI with 496.30 m/z was identical to that of LPC (16:0). LMI, low-mass ion; MS/MS, tandem mass spectrometry; LPC, lysophosphatidylcholine; OVC, ovarian cancer.

In order to identify LMIs with 184.05 and 496.30 m/z, candidate metabolites within ± 0.05 m/z difference were identified using the Human Metabolome Database (HMDB). Ten candidate metabolites with 184.05±0.05 m/z were identified (Table II). Among the candidate metabolites, the metabolic description of HCA in the HMDB was most correlated with OVC, and LPC (16:0) was the only metabolite with 496.30±0.05 m/z (Table II). The LMI with 496.30 m/z on the mass spectrum (Fig. 3A) was further analyzed using MS/MS analysis and was identified to be LPC (16:0) through comparing the MS/MS spectrum of lipid compounds (Fig. 3B).

### Differential level of HCA and LPC (16:0)

**Figure 4:**
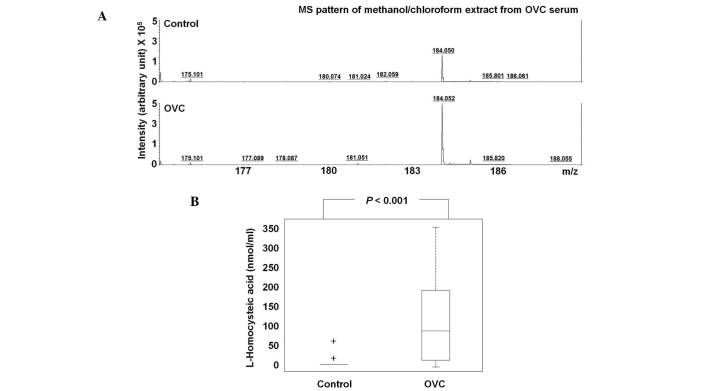
HCA is significantly increased in the sera of the patients with OVC. (A) Mass peak pattern of a low-mass ion with 184.05 m/z on mass spectra. (B) Increase in HCA in patients with OVC compared with the control participants. HCA was not detected in the majority of the control participants. HCA was found to be significantly increased in the sera of the patients with OVC (P<0.001). HCA, L-homocysteic acid; OVC, ovarian cancer; MS, mass spectrometry.

The level of HCA was assessed in 63 control participants and 25 patients with OVC (Table III). Due to insufficient amounts of sera, HCA was not detected in 59/63 of the controls, but the majority of cases of OVC (16/25) exhibited significantly higher levels of HCA, with the mean HCA concentration in the sera of the control individuals being 0.16 nmol/ml compared with 0.60 nmol/ml in the patients with OVC (P<0.001; Fig. 4A). At the cutoff of 10 nmol/ml, the sensitivity and specificity of HCA were 64.0 and 96.9%, respectively; thus, HCA may have potential for OVC screening (Table III).

**Figure 5:**
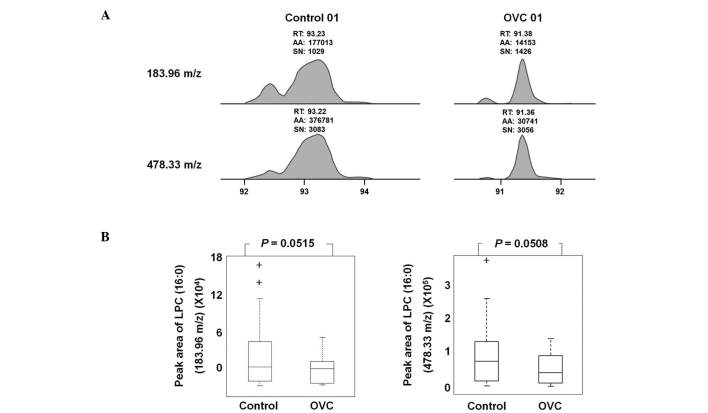
Decreased level of LPC (16:0) in the sera of patients with OVC. The level of LPC (16:0) was assessed through integrating the appropriate peaks for LPC, followed by calculating the ratio of the peak areas. (A) Extracted ion chromatogram of LPC (16:0) in the sera of the control participants and the patients with OVC. Peak areas at 183.96 and 478.33 m/z indicated the concentration of LPC (16:0). (B) The peak area of LPC (16:0) at 183.96 and 478.33 m/z. The peak area of LPC (16:0) with either 183.96 or 478.33 m/z was decreased in the sera of the patients with OVC compared with the controls, but was not significantly different (P=0.0515 and P=0.0508, respectively). LPC, lysophosphatidylcholine; OVC, ovarian cancer; RT, retention time; AA, peak area counts; SN: signal-to-noise ratio.

LPC (16:0) was detected as an LMI with either 183.96 or 478.33 m/z in LC-MS/MS analysis (Fig. 5A). A sufficient amount of sera was obtained from 19 control individuals and 20 patients with OVC to quantify the level of LPC (16:0) and peak areas of 183.96 and 478.33 m/z were determined (Table IV). The peak area was variable depending on the individual samples, but the level of LPC (16:0), represented by peak areas of 183.96 and 478.33 m/z, was observed to be lower in the sera of patients with OVC compared with that of the controls (P=0.0515 and 0.0508, respectively; Fig. 5B).

### Clinicopathological relevance of LPC (16:0) and HCA in OVC

Increased LPC (16:0) was found to be significantly correlated with tumor grade (P=0.045). Although not statistically significant, possibly due to the small number of samples, HCA and LPC (16:0) were found to be correlated with stage and tumor histology (data not shown).

## Discussion

Despite previous investigations, a diagnostic marker for the early diagnosis of OVC has yet to be elucidated. Previous markers which have been used for OVC, including CA125 and HE4, only detected OVC at the late stages of cancer development and lacked efficiency during early tumor growth (13,14).

Metabolic compounds are detected as LMIs in mass spectrometry. Our previous study showed an example of LMI profiling for cancer screening (15). However, at present, the dynamic status of metabolic compounds in the blood is poorly understood. Metabolic compounds in the blood are capable of showing disease status; therefore, profiling LMIs may be useful not only for understanding cancer, but also for identifying biomarkers. Furthermore, recent mass technology, including MALDI-TOF and liquid chromatography-MS/MS, has been found to provide extremely precise and accurate data on LMIs. Therefore, the present study aimed to profile LMIs in serum extracts to assess whether such profiling is capable of discriminate OVC. PCA-DA results showed that the profile of LMIs discriminated OVC (Fig. 1). Only one control case was assigned as OVC over the six experimental repeats (Fig. 1), allowing the LMIs with a significant effect of discriminating OVC to be selected (Fig. 2). Two metabolic compounds were identified and quantified: HCA and LPC (16:0) (Figs. 3–5).

HCA has been reported to affect the oxidation of homocysteinethiolactone to sulfated glycosaminoglycans in cartilage (16). The free base of homocysteinethiolactone has been found to induce carcinogenesis in a mouse model, thus abnormal homocysteine metabolism may be associated with carcinogenesis (16). Dysregulated levels of HCA have not been reported in cancers, although markedly increased HCA has been detected in the cerebrospinal fluid of patients with lymphoma treated with methtrexate (17,18). In the present study, the profiling of LMIs revealed that the level of HCA was different in the serum of patients with OVC compared with healthy control individuals, which was shown through the quantification of HCA in the sera of the controls and the patients with OVC (Fig. 4 and Table III). HCA was not detected in the majority of the control participants, but many of the patients with OVC (16/25) showed significantly higher HCA levels (Table III). At the cutoff of 10 nmol/ml, the sensitivity and specificity of HCA were 64.0 and 96.9%, respectively. The biological implications of upregulated HCA in the sera of patients with OVC has yet to be elucidated and the level of HCA in other types of cancer has yet to be reported. However, the present study found that HCA has strong potential for OVC screening.

The level of LPC in the blood of patients with cancer varies depending on the type of cancer, with LPC found to be decreased in breast cancer (19) and increased in hepatocellular carcinoma (20). In the present study, LPC (16:0) was observed to be decreased in the serum of patients with OVC (Fig. 5). LPC acts as a bioactive mediator in wound healing and inflammation (21), but also has a role in the progression of OVC (22) and lung cancer (23). LPC has many subtypes, and each subtype has a different length of carbon chain. Although the role of each LPC subtype has yet to be elucidated, in the present study, LPC (16:0) was found to be correlated with tumor grade in patients with OVC (P=0.045).

In conclusion, the present study demonstrated that LMI profiling may be a powerful tool to obtain valuable data on metabolic compounds, as well as to identify biomarkers for cancer screening. Despite the lack of explanation for the pathological changes in HCA and LPC (16:0) in the sera of patients with OVC, the findings of the present study demonstrate that HCA is a powerful serological biomarker for OVC screening. In the present study, LPC alone was not helpful to increase the discriminating power of HCA; however, with the identification of other candidate metabolites in the future, HCA has the potential to be used in multi-biomarker OVC screening.

## Figures and Tables

**Table I:** Characteristics of the patients with ovarian cancer and the control participants included in the present study.

Parameter	Ovarian cancer (n=142)	Control (n=100)
Age, mean ± SD	52±13	51±13
Stage, n (%)		
IA	37 (26.1)	-
IB	2 (1.4)	-
IC	12 (8.5)	-
IIA	0 (0.0)	-
IIB	1 (0.7)	-
IIIA	0 (0.0)	-
IIIB	1 (0.7)	-
IIIC	77 (54.2)	-
IV	12 (8.5)	-
Histology, n (%)		
Serous	90 (63.4)	-
Mucinous	23 (16.2)	-
Endometrioid	8 (5.6)	-
Clear cell	11 (7.7)	-
Transitional cell	7 (4.9)	-
Mixed	3 (2.1)	-
Grade, n (%)		
Mild	24 (16.9)	-
Moderate	35 (24.6)	-
Severe	83 (58.5)	-

SD, standard deviation.

**Table II:** Candidate metabolites with H^+^ adducts in human metabolome database.

Compound	Name	Adduct	Adduct MW (Da)	Compound MW (Da)	Delta
Metabolites with 184.05±0.05 m/z					
HMDB00017	4-Pyridoxic acid	M+H	184.06043	183.05316	0.010434
HMDB02205	L-Homocysteic acid	M+H	184.02742	183.02014	0.022581
HMDB11657	2,6-Diamino-4-hydroxy-5-N- ethylformamidopyrimidine	M+H	184.08290	183.07563	0.032901
HMDB33141	2-Amino-α-carboline	M+H	184.08692	183.07965	0.036923
HMDB29723	Saccharin	M+H	184.00629	182.99901	0.043710
HMDB02832	Methylnoradrenaline	M+H	184.09682	183.08954	0.046819
HMDB15652	Levonordefrin	M+H	184.09682	183.08954	0.046819
HMDB00819	Normetanephrine	M+H	184.09682	183.08954	0.046819
HMDB29455	Ginkgotoxin	M+H	184.09682	183.08954	0.046819
HMDB00068	Epinephrine	M+H	184.09682	183.08954	0.046819
Metabolites with 496.30±0.05 m/z					
HMDB10382	LPC (16:0)	M+H	496.33977	495.33249	0.039765

LPC, lysophosphatidylcholine.

**Table III:** L-homocysteic acid levels in the sera of 63 control participants and 25 patients with OVC.

Control						OVC	
	
Sample no.	Conc. (nmol/ml)	Sample no.	Conc. (nmol/ml)	Sample no.	Conc. (nmol/ml)	Sample no.	Conc. (nmol/ml)
Control 01	0.000	Control 26	0.000	Control 51	0.000	OVC 01	25.991
Control 02	0.000	Control 27	0.000	Control 52	0.000	OVC 02	0.000
Control 03	0.000	Control 28	0.000	Control 53	48.750	OVC 03	109.620
Control 04	0.000	Control 29	0.000	Control 54	0.000	OVC 04	0.000
Control 05	0.000	Control 30	0.000	Control 55	0.000	OVC 05	20.037
Control 06	0.000	Control 31	0.000	Control 56	0.000	OVC 06	0.000
Control 07	0.000	Control 32	0.000	Control 57	0.000	OVC 07	0.000
Control 08	0.000	Control 33	0.000	Control 58	0.000	OVC 08	0.000
Control 09	0.000	Control 34	0.000	Control 59	0.000	OVC 09	116.759
Control 10	0.000	Control 35	0.000	Control 60	0.000	OVC 10	79.676
Control 11	0.000	Control 36	0.111	Control 61	0.000	OVC 11	61.083
Control 12	0.000	Control 37	0.000	Control 62	0.000	OVC 12	172.352
Control 13	0.000	Control 38	0.000	Control 63	0.000	OVC 13	286.398
Control 14	0.000	Control 39	0.000			OVC 14	203.306
Control 15	0.000	Control 40	0.000			OVC 15	0.000
Control 16	0.000	Control 41	0.000			OVC 16	175.713
Control 17	0.000	Control 42	0.000			OVC 17	175.676
Control 18	0.000	Control 43	0.000			OVC 18	74.824
Control 19	4.981	Control 44	0.000			OVC 19	133.407
Control 20	0.000	Control 45	49.750			OVC 20	344.787
Control 21	0.000	Control 46	0.000			OVC 21	206.537
Control 22	0.000	Control 47	0.000			OVC 22	0.000
Control 23	0.000	Control 48	0.000			OVC 23	0.000
Control 24	0.000	Control 49	0.000			OVC 24	72.565
Control 25	0.000	Control 50	0.000			OVC 25	0.000

OVC, ovarian cancer; Conc., concentration.

**Table IV:** Level of peak area in the sera of 19 control participants and 25 patients with OVC.

Control			OVC		
	
Sample no.	183.96 m/z	478.33 m/z	Sample no.	183.96 m/z	478.33 m/z
Control 01	177013	376781	OVC 01	14153	30741
Control 02	69756	137502	OVC 02	5859	13745
Control 03	124532	272566	OVC 03	7971	15462
Control 05	25420	66997	OVC 04	8396	19590
Control 06	54801	128622	OVC 05	20228	53475
Control 07	37451	84449	OVC 06	16271	39552
Control 08	83913	172936	OVC 07	32559	69217
Control 09	24680	64998	OVC 08	25213	60890
Control 10	24203	53327	OVC 09	12003	30286
Control 11	154157	376840	OVC 10	26121	59037
Control 12	22627	51433	OVC 11	33905	51453
Control 13	48125	102808	OVC 12	51453	115718
Control 14	52038	109721	OVC 13	40846	87236
Control 15	45143	104486	OVC 14	68258	149730
Control 16	10764	24637	OVC 15	49476	114076
Control 17	4301	10910	OVC 16	53280	132663
Control 18	6538	15969	OVC 17	49151	109521
Control 19	5664	8595	OVC 18	40636	84156
Control 20	1894	3979	OVC 19	35516	78462
			OVC 20	59084	130141
			OVC 21	38533	80979
			OVC 22	14389	31289
			OVC 23	7779	15915
			OVC 24	35065	83313
			OVC 25	12879	29815

OVC, ovarian cancer.
